# Supplementary material for: Effect of Kinases in Extracellular Vesicles from HIV-1-Infected Cells on Bystander Cells
Source: Cells. 2025 Jan 15;14(2):119. doi: 10.3390/cells14020119 (PMC11763833; doi:10.3390/cells14020119)
Supplement: Supplementary file 1 [file cells-14-00119-s001.zip › Mensah et al. Supplementary Materials.pdf]

Supplementary Materials:

Effect of Kinases in Extracellular Vesicles from HIV-1-Infected Cells on Bystander Cells

Gifty A. Mensah[1], Anastasia Williams[1], Pooja Khatkar[1], Yuriy Kim[1], James Erickson[1], , Alexandra Duverger[2], Heather Branscome[1], Kajal Patil[1], Hafsa Chaudhry[1], Yuntao Wu[3], Olaf Kutsch[2], and Fatah Kashanchi [1,*]

1 Laboratory of Molecular Virology, George Mason University, Manassas, VA 20110.
2 Department of Medicine, University of Alabama at Birmingham, Birmingham, AL 35294.
3 National Center for Biodefense and Infectious Diseases, School of Systems Biology, George Mason University, Manassas, VA, 20110.
* Correspondence: Fatah Kashanchi, Ph.D., Laboratory of Molecular Virology, George Mason University, Discovery Hall Room 182, 10900 University Blvd., Manassas, VA 20110, USA, Tel.: 703-993-9160, Fax: 703-993-7022, Email: fkashanc@gmu.edu

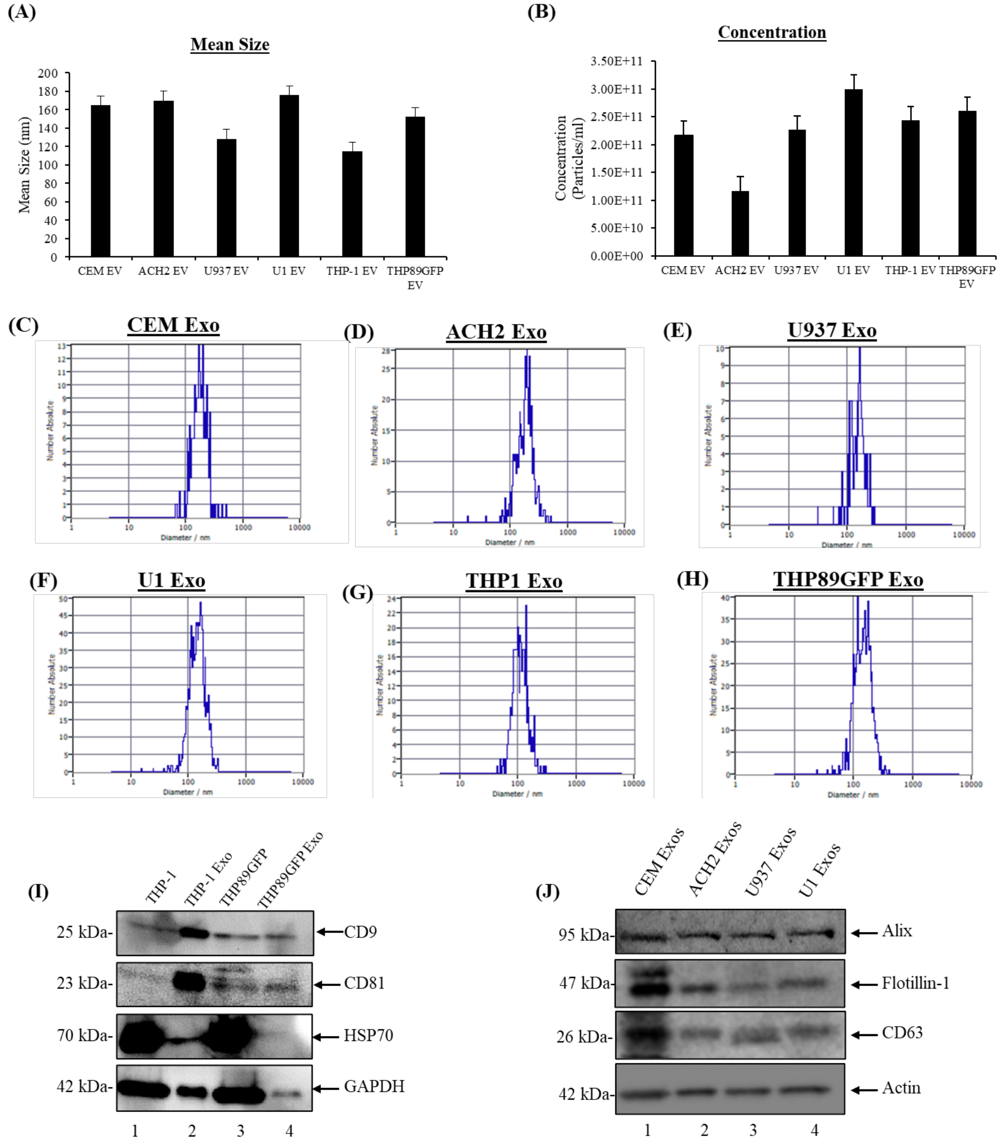

Figure S1. Characterization of exosomes isolated via differential ultracentrifugation. Exosomes (Exo) were isolated via differential ultracentrifugation. The 100K population isolated from CEM, ACH2, U937, U1, THP-1, and THP89GFP cells were analyzed via Nanoparticle Tracking Analysis (NTA) for (**A**) size distribution (average) and (**B**) number of total vesicles released (average), as well as peak distribution (**C-H**). To further confirm Exo populations, exosome markers (CD9, CD81, HSP70, Alix, Flotillin-1, and CD63) were assessed via Western blot (**D-J**).

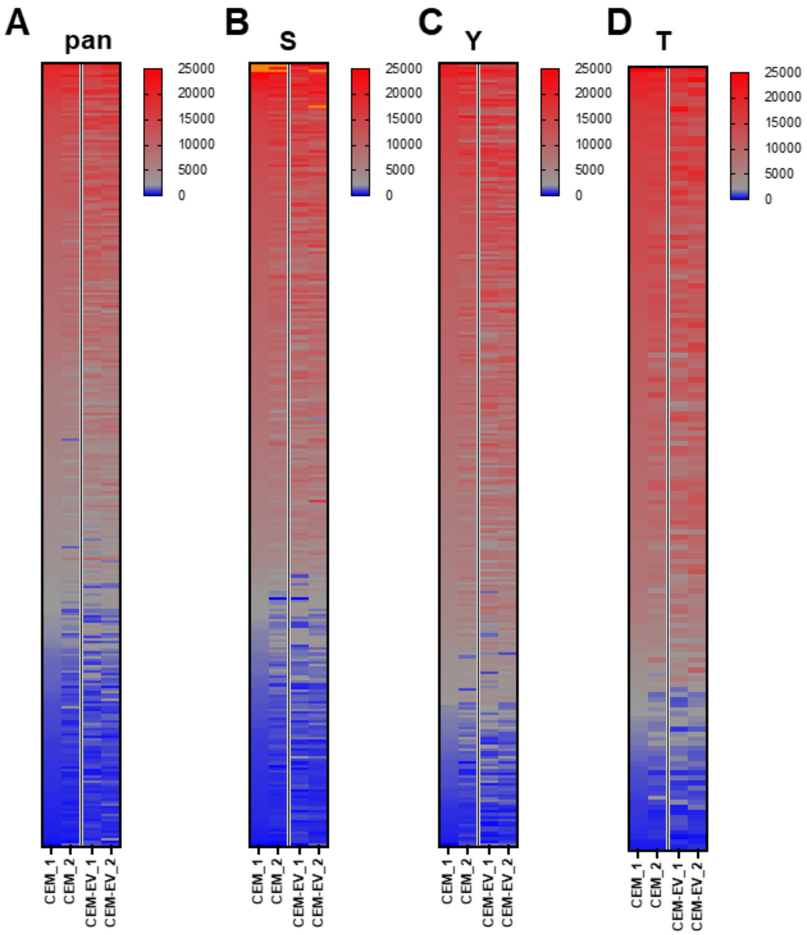

Figure S2. Antibody array data comparing the protein content and the protein phosphorylation patterns between cell and exosomes. The protein content and protein phosphorylation patterns of CEM T-cells and CEM cell-produced exosomes shown in Figure 1C were resolved for pan-specific, as well as serine-, tyrosine- and threonine-specific phosphorylation signals to demonstrate that the finding that exosomes are largely a phospho-proteomic phenocopy of the producer cells applies for pan-specific and phosphorylation targets. Signals that exceed the range are indicated in orange. Each antibody on the array is present in duplicates and the individual signals obtained for the analysis of CEM cells and CEM-produced exosomes are shown as CEM _1/CEM_2 or CEMEV_1/CEMEV_2, respectively.
